# Two swimming modes in Trachymedusae; bell kinematics and the role of giant axons

**DOI:** 10.1242/jeb.239830

**Published:** 2021-05-25

**Authors:** Matthew E. Meech, Claudia E. Mills, Steven H. D. Haddock, Robert W. Meech

**Affiliations:** 1 BBC Natural History Unit, Whiteladies Road, Bristol BS8 2LR, UK; 2 Friday Harbor Laboratories, 620 University Road, Friday Harbor, WA 98250, USA; 3 Monterey Bay Aquarium Research Institute, 7700 Sandholdt Road, Moss Landing, CA 95039, USA; 4 School of Physiology, Pharmacology and Neuroscience, University of Bristol, Bristol BS8 1TD, UK

**Keywords:** Cnidaria, Dual swimming, Giant motor axon, Hydromedusae, Kinematics, Phylogenetic tree

## Abstract

Although members of the Rhopalonematidae family (Cnidaria, Hydrozoa, Trachymedusae) are known to exhibit unusually powerful jet swimming in addition to their more normal slow swimming behaviour, for the most part, reports are rare and anecdotal. Many species are found globally at depths of 600–2000 m, and so observation and collection depend on using remotely operated submersible vehicles. With a combination of *in situ* video footage and laboratory measurements, we have quantified kinematic aspects of this dual swimming motion and its electrophysiology. The species included are from two Rhopalonematidae clades; they are *Colobonema sericeum*, *Pantachogon haeckeli*, *Crossota millsae* and two species of *Benthocodon.* Comparison is made with *Aglantha digitale*, a species from a third Rhopalonematidae clade brought to the surface by natural water movement. We find that although all Rhopalonematidae appear to have two swimming modes, there are marked differences in their neural anatomy, kinematics and physiology. Giant motor axons, known to conduct impulses during fast swimming in *A. digitale*, are absent from *C. sericeum* and *P. haeckeli*. Slow swimming is also different; in *C. sericeum* and its relatives it is driven by contractions restricted to the base of the bell, whereas in *A. digitale* it is driven by contractions in the mid-bell region. These behavioural differences are related to the position of the different clades on a ribosomal DNA-based phylogenetic tree. This finding allows us to pinpoint the phylogenetic branch point leading to the appearance of giant motor axons and escape swimming. They place the remarkable dual swimming behaviour of members of the Rhopalonematidae family into an evolutionary context.

## Abstract (precis)

**Summary:** 18S ribosomal DNA data support anatomical, kinematic and electrophysiological evidence that identifies the phylogenetic branch point giving rise to giant-axon-based fast and slow swimming in the Rhopalonematidae.

## INTRODUCTION

The phylum Cnidaria contains a diverse collection of life forms exhibiting a wide variety of behaviours. Some adult forms, such as the hydroids, sea anemones and corals, are sessile or remain so for long periods. By contrast, the Medusozoa are either fully motile or have a motile stage in their life cycle. Four classes of jellyfish make up the Medusozoa: the Scyphozoa, the Cubozoa, the Staurozoa and the Hydrozoa. Included in the Hydrozoa are several orders of jellyfish such as the Trachymedusae. Here, we examine swimming in a specific trachymedusan family, the Rhopalonematidae. This family is unique among jellyfish in having a dual system of swimming.

The most extensive comparative description of medusan swimming is that of Gladfelter (1972, 1973), who picked out the trachymedusa *Aglantha digitale* as being of particular note because it gives “a single powerful contraction in response to a probe, which is considerably more effective than the normal type”. He suggested that this powerful contraction may be a means by which *Aglantha* can escape from a predator. In *Aglantha*, this substitutes for the slow ‘crumpling’ response of most hydromedusae to any form of threat (Hyman, 1940). During escape, a single contraction propels *Aglantha* by five body lengths with a maximum velocity of ∼40 cm s^−1^ (Donaldson et al., 1980; Meech, 2015), markedly more than the values reported by Gladfelter (1973) for other medusae. These range from an average of 9 cm s^−1^ (*Gonionemus vertens*) to 2.5 cm s^−1^ (*Bougainvillia multitentaculata*). The exceptional maximum velocity of *A. digitale* is associated with the presence of two sets of giant axons, which are responsible for conducting excitation around and up the animal so that the entire musculature contracts within 100 ms. This violent contraction means that the base of the bell-shaped body forms a nozzle through which the bell's water contents are expelled at high velocity (Ford and Costello, 2000).

Fast escape swims of this kind are energetically expensive and relatively rare. As a way of more gradually gaining height in the water column, *Aglantha* performs a weaker form of swimming (slow swimming). During a single slow swim, the animal moves forward ∼15 mm, about one body length (Mackie, 1980), thrust being minimized (and energy conserved) because the base of the bell remains wide open (Meech, 2015). The clear distinction between fast and slow swimming extends beyond the large difference in power to the myoneural basis of swimming, and it seems appropriate to describe the form of propulsion used by *Aglantha* as dual swimming.

The Trachymedusae, together with three other hydrozoan orders – the Narcomedusae, the Limnomedusae and the Actinulida, are sometimes classed together as the Trachylina. This broad classification, based largely on anatomical and developmental considerations, has required fine-tuning as a consequence of recent DNA analysis (Collins et al., 2008). Such a readjustment applies to the Rhopalonematidae, which hitherto have been regarded as a single, albeit loosely connected, trachymedusan family (Collins et al., 2008; Lindsay et al., 2017). DNA analysis now suggests that this supposedly single unit is in fact made up of three major groups: (1) the *Crossota* group; (2) the *Aglantha*, *Aglaura* and *Amphogona* subclade (here called the Aglaurinae); and (3) the *Colobonema*, *Pantachogon*, *Rhopalonema* subclade (here called the Rhopaloneminae).

Other members of the Aglaurinae, *Aglaura hemistoma* and *Amphogona apicata*, have been observed to perform two distinct modes of swimming like *A. digitale* (Mills et al., 1985; Wrobel and Mills, 1998). *Amphogona* is also known to have giant motor axons that run radially inside the bell from the margin to the apex (Mills et al., 1985). There is also a ring giant axon running round the base of the bell and a large axon that runs down the side of each tentacle, just as in *Aglantha*. Another common feature is their streamlined bullet, or prolate, shape.

Members of the other Rhopalonematidae clades are less well studied because they occur at depths of 200–3000 m and are rarely brought to the surface. Nevertheless, it has been known anecdotally for some years that they exhibit unusually powerful swims. In *Ransonia krampi*, the swim is “strong enough to propel it out of a shallow container and onto the lab bench” (Mills et al., 1996). According to Gladfelter (1973), swimming in *Pantachogon haeckeli* is “the most powerful seen in any medusa (other than the Siphonophores)”. In a single contraction, the initially motionless medusa (∼1 cm high) shoots ∼10 cm across the container. Although it is tempting to assume that all of these fast swims represent escape behaviour, this is by no means certain because it is not known whether they are elicited by specific external stimuli. Here, we find that freshly captured specimens of *Pantachogon haeckeli* and *Colobonema sericeum* are highly vibration sensitive and that, unlike the Aglaurinae, their fast swims do not depend on giant axons. We find that members of the Rhopaloneminae and Crossota clades are also capable of slow swimming, but that, unlike *Aglantha*, these contractions are confined to the bell margin. Thus, although all three Rhopalonematidae clades exhibit dual swimming, the myoneural basis is different. These findings allow us to pinpoint the phylogenetic branch point leading to these differences in organization.

Previous examinations of independently evolved behaviours within a clade provide important insights into their neural basis. Swimming has evolved multiple times in the nudipleura molluscs, for example. In some species, the swimming pattern is generated by entirely different sets of neurons; in others, homologous neurons play similar roles (Katz, 2011; Newcomb et al., 2012). Here, we do not explore differences in generating the rhythmicity of swimming, but instead focus on the physiology and mechanics of the movement itself.

## MATERIALS AND METHODS

*Aglantha digitale* (O. F. Müller 1776) specimens were collected from surface water at the dock of Friday Harbor Laboratory, Friday Harbor, WA, USA. They were transferred to a narrow transparent chamber filled with natural seawater at 10°C. Swimming, which was confined to a narrow focal plane, was video recorded against a black background at 300 frames s^−1^. Escape swims were elicited with a glass probe.

Specimens of other Rhopalonematidae clades were collected from sites within Monterey Bay, CA, USA, at depths and temperatures that ranged from 200 to 600 m and from 6 to 7°C. The remotely operated submersible vehicle (ROV) Ventana lowered from the Monterey Bay Aquarium Research Institute (MBARI) vessel R/V Rachel Carson and equipped with a multiple-bin suction sampler (Youngbluth, 1984) was used to collect seven specimens of *Colobonema sericeum* E. Vanhöffen 1902 and a single *Pantachogon haeckeli* O. Maas 1893. In the laboratory, the animals were bathed with natural seawater collected at the appropriate depth and were kept in the dark at 6°C.

Swimming behaviour of specimens of *Colobonema* and *Crossota millsae* E. V. Thuesen 2003 in their natural environment were filmed *in situ* with on-board cameras. For more details, see Robison (1993) and Matsumoto et al. (2020). Bell diameter data for *Benthocodon hyalinus* (Larson et al., 1990) were obtained by measurement from videos provided by Elliott et al. (2017; NOAA Ship Okeanos Explorer). Data for *Benthocodon pedunculatus* (H. B. Bigelow 1913) were measured from the supplementary material provided in Matsumoto et al. (2020; ROV Tiburon dive #894).

To discover whether *Colobonema* or *Pantachogon* can swim normally in the laboratory, despite experiencing the large pressure change upon being brought to the ocean surface (up to 6.2 MPa), specimens were filmed on a cooled microscope stage (11–16°C). In the case of *Pantachogon*, the whole animal was small enough to be tethered and stimulated with a bipolar electrode. *Colobonema* specimens were too large to be treated in this way. However, the bell is made up of octants of muscle epithelium (myoepithelium) outlined by the eight radial canals that run from the margin to the bell apex. Sections of body wall consisting of an octant and its associated radial canals were pinned out in a Sylgard-coated dish, subumbrella side upward, using either steel pins or spines from the cactus *Opuntia*. In each case, a bipolar stimulating electrode was placed so as to lightly touch the myoepithelium without damaging it, and a 2 ms electrical stimulus was adjusted to be just supra-threshold.

### Image analysis

Video recordings were converted to a series of sequential frames. Bell dimensions were measured using the image-analysis program ImageJ (see Schneider et al., 2012) to provide an estimate of the duration and extent of the swimming bell contraction. To measure individual radii, the image was separated into its two halves by erecting a line at right angles from the base passing through the apex of the bell. Some images were adjusted using Adobe Photoshop software simply to enhance the contrast and remove the background.

### Molecular phylogeny

Sequences from 18S ribosomal DNA were obtained in the laboratory or via GenBank (Collins et al., 2008; Lindsay et al., 2017). They were aligned and trimmed using GUIDANCE2 (Sela et al., 2015) with MAFFT (Katoh and Standley, 2013) as the sequence-alignment program. The resulting dataset with low-support columns removed was used with IQTREE-version 1.x (Minh et al., 2020) to generate a consensus tree using the options –nt AUTO (to determine the best number of separate processing threads) and –lbp 1000 to specify the number of replicates using a local bootstrap probability method.

## RESULTS

### Relationships between different trachyline species

**Fig. 1.**
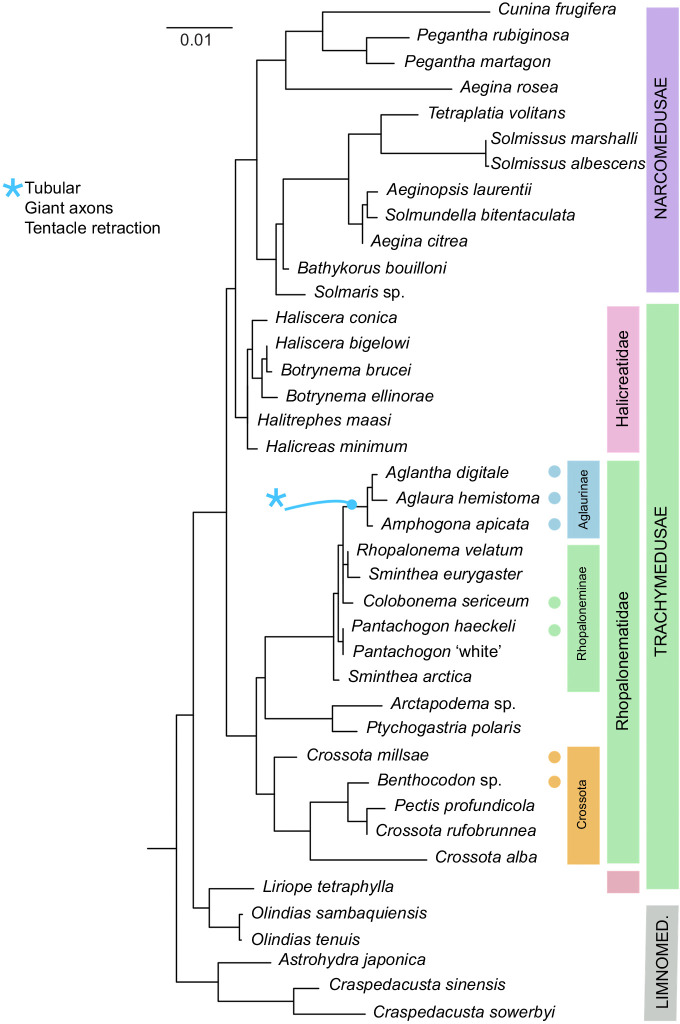
**Relationships between different trachyline species.** Phylogenetic tree based on 18S ribosomal DNA data; bootstrap values omitted for clarity. Filled circles indicate the species referred to in the text. The lineage leading to the Aglaurinae is marked with a blue asterisk; it signifies the appearance of giant motor axons, a more tubular body shape and fast tentacle retraction, see Discussion. Bar length indicates 0.01 substitutions per site.

Fig. 1 shows a phylogenetic tree for selected species of Trachymedusae, Narcomedusae and Limnomedusae based on 18S ribosomal DNA data from species available via GenBank plus new data obtained from species collected locally in Monterey Bay. The Trachymedusae are shown separated into the Rhopalonematidae and the Halicreatidae. A previous DNA-based examination of the Rhopalonematidae has supported its division into three main clades (Collins et al., 2008), and in Fig. 1 they are named as the Crossota group, the Aglaurinae and the Rhopaloneminae. Their relative positions are shown together with the positions of two members of a fourth clade, the Ptychogastriidae. Filled circles highlight the species used to provide the new analysis reported here.

### Observations of specimens in their natural environment

**Fig. 2.**
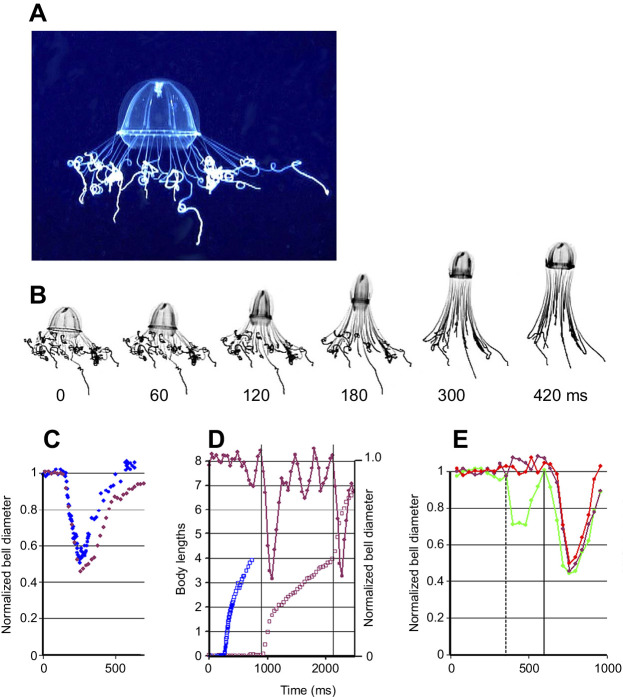
**Forms of swimming by *Colobonema sericeum* in its natural environment.** (A) A resting specimen with tentacles deployed to catch prey. (B) Frames from a video recording of a fast swim; frame intervals below. The outside of the bell is almost hemispherical at rest, but the distribution of the mesogloea means that the subumbrellar musculature is more cone shaped. During contraction, the bell becomes more tubular and the tentacles more extended. Bell refilling is incomplete even at 420 ms. (C) Change in bell diameter measured for *C. sericeum* (filled maroon circles; change measured at the base of bell) and *Aglantha digitale* (filled blue circles; change measured mid-way up the bell) during a fast swim. All data points normalized to the diameter at rest. (D) Forward movement of *C. sericeum* during a swim sequence (open maroon circles; left axis) and associated changes in bell diameter (filled maroon circles; right axis). The sequence begins with a slow swim, which has no perceptible effect on forward movement; a fast swim (shown by the first vertical line) causes the animal to move forward by about two body lengths; then there are three slow swims before the second fast swim (shown by the second vertical line). Also shown is the forward movement of *A. digitale* during a single fast swim (open blue circles). (E) *Colobonema* *sericeum* changes in bell dimensions during near-horizontal swimming. A slow swim (dashed vertical line shows the start) can be compared with the fast swim (solid vertical line shows the start) that followed it. The animal was pointing at an angle of +10 to +13 deg from the horizontal throughout the slow swim. Bell apex pointed left. Upper radius (filled green circles) and lower radius (filled red circles) are normalized to the value at rest. The normalized diameter at a point mid-way up the bell (filled maroon circles) remained constant during the slow swim, but its contraction during the fast swim was the same as at the bell base.

Studied in its natural environment using the ROV Ventana, *C*. *sericeum* (Fig. 2A) exhibits bursts of powerful swimming as if evading capture. However, it is unclear whether the swims are elicited in response to water disturbance created by the ROV or because of the bright light used for filming. As noted by previous authors, the animal was likely to lose some or many of its tentacles during high-frequency fast swims (see Movie 1), which suggests that it is normally a relatively rare occurrence. Although we have not found the released tentacles to be luminescent, they may nonetheless function to distract and discourage potential pursuers (Wrobel and Mills, 1998). The remaining tentacles, unlike tentacles in *Aglantha* (Mackie et al., 1989), were found to be unciliated.

Fig. 2B shows one of a series of strong swims exhibited by *C. sericeum* filmed *in situ* (depth, 225 m). It consists of six non-consecutive frames 60–120 ms apart. The time to maximum contraction varied from 120 to 180 ms, with the first of the series of swims being stronger and taking longer to reach its peak. Subsequent contractions often occurred before the bell had fully refilled and the time to peak was shorter. The contraction during a single strong swim, estimated from the change in bell diameter, is plotted in Fig. 2C (filled maroon circles), with measurements from an *Aglantha digitale* escape swim for comparison (filled blue circles). The immediate forward movement was about two body lengths, which is much the same as in *A. digitale* (see Fig. 2D), but *C. sericeum* took longer to get to the four body lengths mark. Unlike *A. digitale*, which contracts its tentacles as the swim is initiated, *C. sericeum* drags its extended tentacles along behind it (Fig. 2B).

Fig. 2D shows that *C. sericeum*, like *A. digitale*, exhibits both fast and slow swims. However in *C. sericeum*, the slow swims are confined to contractions at the base of the bell (bell margin) and there is little or no contraction in the mid-bell region (Fig. 2E), which is what constitutes slow swimming in *A. digitale* (Meech, 2015). When *C. sericeum* is slow swimming, the bell margin may contract asymmetrically. This is seen in Fig. 2E, which plots changes in bell diameter and separate radii for the last in a series of slow swims, followed by a fast swim. A dashed vertical line indicates the start of the slow swim; a solid vertical line shows the start of the fast swim. During the slow swim, the bell contraction occurred on the right hand side, upper radius only (the bell apex was pointing to the left). The margin on the left hand side did not contract, and the diameter measured mid-way up the bell was also unchanged.

**Fig. 3.**
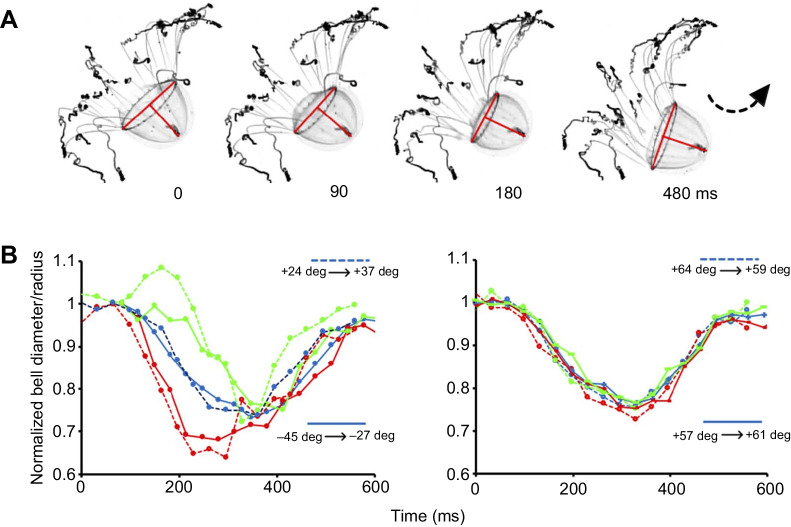
***Colobonema sericeum* changes in bell dimensions during slow swimming.** (A) Frames showing a specimen turning during a slow swim; frame intervals below. The bell apex moves counterclockwise and the superimposed red lines, drawn at right angles, show the bell height, diameter and radii. Note the asymmetrical nature of the contraction at the bell margin. (B) The radius of the bell margin normalized to the value at rest; green circles, right radius; red circles, left radius; with bell apex upward. Also shown is the normalized bell diameter (blue circles). Dashed and solid data lines signify different swims. Bell apex on the right of the margin in each case. Left: data from two asymmetrical slow swims. Animals were oriented +24 and −45 deg from the horizontal, as indicated on the figure. During the contractile phase of each swim, the animal moved counterclockwise by 13 and 18 deg, as noted. Right: data from two symmetrical slow swims. Animals were more vertically oriented (+64 and +57 deg from horizontal, as indicated on the figure). During each swim, the animals changed orientation by 5 and 4 deg, as noted.

In Fig. 2E, the swim was markedly asymmetrical but caused no more than 3 deg change in the animal's orientation. However, on other occasions, the contractile phase of an asymmetrical swim produced a 13–18 deg change in orientation. Fig. 3A shows video frames captured at the end of a series of fast swims when the animal was drifting downward. The slow swim produced a partial correction in the downward drift. As the red lines indicate, the greatest difference between the inside and outside of the turn at the base of the bell occurred at 90 ms. A preliminary analysis of slow swims captured from animals oriented at different angles is shown in Fig. 3B. This suggests that swims at +57 to +64 deg from the horizontal are broadly symmetrical and generate little change in orientation. Swims in animals oriented at +24 to −45 deg from the horizontal show marginal contractions that are initially asymmetric. In these animals, the lower half of the bell contracts only after a ∼100 ms delay when the animal's orientation has moved counterclockwise.

### *Colobonema sericeum* – electrical stimulation of a pinned preparation

The collected specimens of *C. sericeum* were ∼30 mm high and had a semi-transparent body wall, which was ∼3 mm thick. For the first 48 h after capture, specimens would swim vigorously in response to a disturbance of the water surface but they showed no sensitivity to light. They also exhibited periodic sequences of slow swims even without stimulation. After 4 days in the laboratory, *C. sericeum* would respond to electrical stimuli from a superficially placed bipolar electrode but showed no sensitivity to vibration. At no stage did they show the kind of crumpling response to trauma that is observed in many other hydromedusae (Hyman, 1940).

**Fig. 4.**
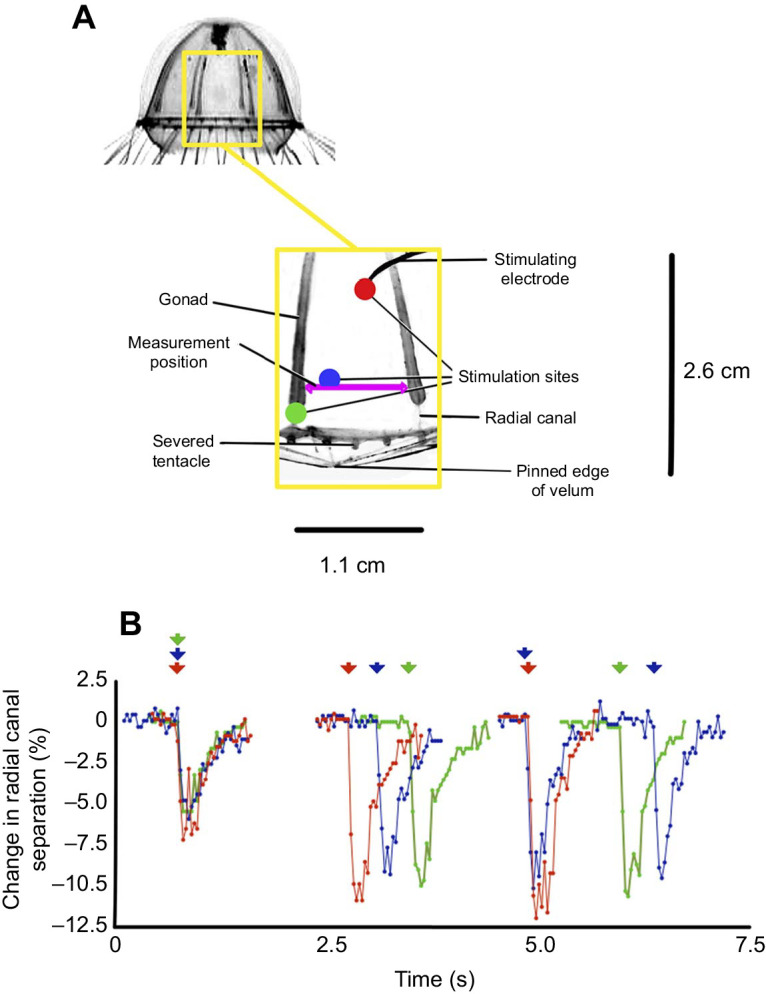
**Fictive swimming in a *C.sericeum* pinned preparation.** (A) Inset: the bell of the intact specimen showing the area to be dissected out and pinned. Main: diagram of the preparation adapted from a video frame, showing stimulus sites on the myoepithelium between two radial canals. The purple arrow-headed line shows the position of the measurements. Gonads cover the canals over most of their length. The apex of the bell has been removed. (B) Plot of the distance between the radial canals during a series of electrical stimuli (arrowheads). The colour of the three sequences corresponds to the colour of the stimulating position marked in A. Onsets of the first contraction in each sequence are aligned.

A number of sites on the subumbrella surface of a pinned preparation were stimulated using the bipolar electrode. Contractions were video recorded through a dissecting microscope and sequences were analysed frame by frame. The bell contains eight radial canals, a large part of each one being covered by an attached gonad. The transparency of the bell means that all eight sets of gonads are visible in Fig. 4A (inset). The experimental preparation consisted of a single octant dissected from the bell and pinned as shown (yellow box). In the space between the end of the gonad and the bell margin, the radial canal is visible but there is no giant axon. The stumps of five tentacles are present at the base of the preparation. The separation of the radial canals was measured at a site near the base of the gonads (shown by a purple arrow-headed line). The change in the separation is plotted in Fig. 4B for three positions of the stimulating electrode. Contractions reached their peak in ∼100 ms as they did during swimming in the wild (Fig. 2). In both cases, bells recovered to the resting state in ∼800 ms.

The first electrical stimulus in a series produced a 5–7% reduction in the separation of a single pair of radial canals in the pinned preparation (Fig. 4B), considerably less than the change in diameter of the free-swimming animal shown in Fig. 2. In Fig. 4B, subsequent stimuli of the pinned preparation produced a greater contraction, the radial canal separation decreasing by ∼10%, but an increased contraction is not evident in the whole animal shown in Fig. 2D. Measurements of radial canal separation nearer to the bell apex showed that the movement was smaller here (not shown). This was the case even when the stimulating electrode was located in the upper half of the bell. Thus, the myoepithelium responds to electrical stimulation, even in apical regions where there is little or no visible contraction.

In some jellyfish, the epithelium on the surface of the bell is excitable, and stimulating it can lead to a crumpling defence response (Mackie and Passano, 1968). In order to exclude the possibility that the electrical stimulus was strong enough to excite this exumbrellar epithelium and excite the marginal nerves indirectly, we cut into the subumbrellar myoepithelium, created an isolated rectangular ‘island’ of muscle, and then stimulated it. The myoepithelium within the rectangle contracted but the contraction did not spread outside to the rest of the bell.

### *Pantachogon* – electrical stimulation of the whole animal

Like the *C. sericeum* specimens, the *P.* *haeckeli* specimen would swim vigorously in response to a disturbance of the water surface but showed no sensitivity to light. It also showed no crumpling response to trauma like that observed in many other hydromedusae (Hyman, 1940). However, it responded to touch with a fine probe at the internal surface of the base of the bell, each time giving a strong contraction. It also exhibited periodic sequences of slow swims even without stimulation.

**Fig. 5.**
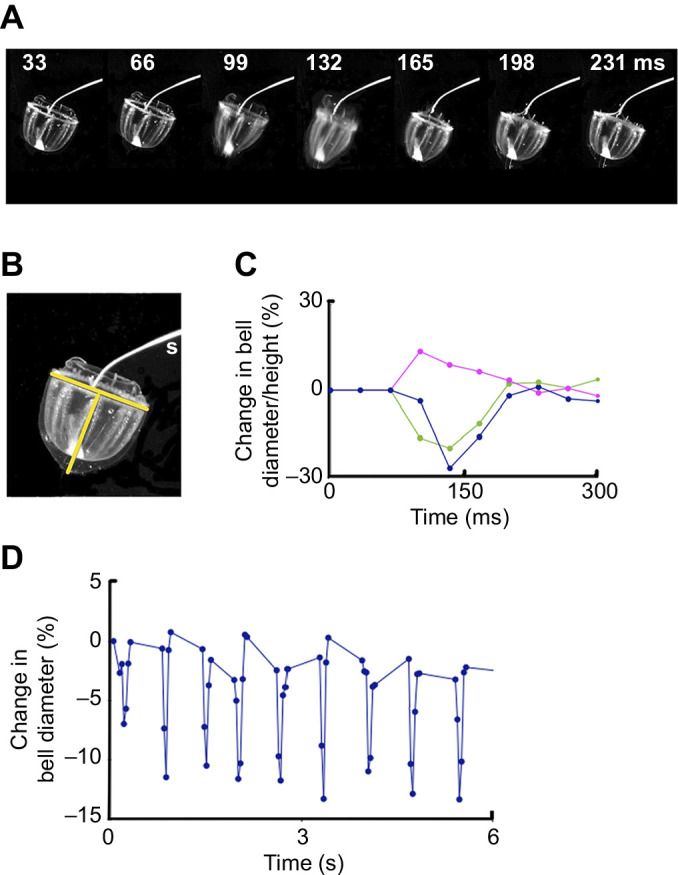
**Electrically stimulated swim in *Pantachogon haeckeli*.** (A) Frames from a video recording showing the effect of 2 ms supra-threshold electrical pulse. The bipolar metal electrode (indicated by ‘s’ in B) was in contact with the myoepithelium, and the animal was tethered to the electrode by one of its tentacles. Bell apex points downwards. Interval between frames, 33 ms. (B) Measurement positions shown by yellow lines. Bell diameter, ∼1.5 cm. (C) Change in bell diameter (%) at the base (blue circles) and mid-bell (green circles) regions; pink circles indicate the change in bell height. (D) Change in bell diameter (base) during a sequence of spontaneous slow swims. Swim frequency, 1.5 Hz.

The *P. haeckeli* specimen was examined under a binocular microscope on a cooled stage, and the base was probed with a bipolar stimulating electrode. Fortuitously, the probe became attached to one of the fine tentacles. This meant that although the specimen was free to contract, its forward movement was restricted, permitting the entire swim movement to be recorded. For Fig. 5A, frames were selected from a video sequence to show the contraction at 33 ms intervals. In Fig. 5B, the stimulating electrode(s) can be seen positioned on the surface of the myoepithelium near the base of the bell. Measurements of the base diameter and the bell height (identified by the yellow lines in Fig. 5B) are shown in Fig. 5C, together with a second diameter measurement mid-way up the bell. There was an almost 30% decrease in the diameter of the base of the bell and a 15% increase in its height. The peak contraction occurred between frame 3 and frame 4 so that the time to peak was 33–66 ms, even shorter than that found in *C. sericeum* or *A. digitale* (∼100 ms in Fig. 2). The total duration of the swim was ∼165 ms, considerably less than that for *C. sericeum*.

The *P. haeckeli* specimen studied by Gladfelter (1973) performed spontaneous sequences of slow swims. Fig. 5D shows that our specimen behaved similarly. Here, the changes in base diameter indicate that the animal produced nine swims in a 6 s period. In each case, the change in bell diameter was less than 15%, i.e. less than half that elicited by an electrical stimulus.

### *Crossota millsae* – tentacle management

There is a marked difference between *A. digitale* and *C. sericeum* in the way they manage their tentacles when performing a fast swim. In *A. digitale*, tentacle contraction is associated with activity in the tentacle giant axon (Roberts and Mackie, 1980) and is completed as the swim gets underway; in *C. sericeum*, the tentacles remain extended throughout the swim (Fig. 2B). We therefore examined tentacle management in the other major subclade of the Rhopalonematidae, the Crossota subclade, the members of which are also known to perform powerful swimming movements.

**Fig. 6.**
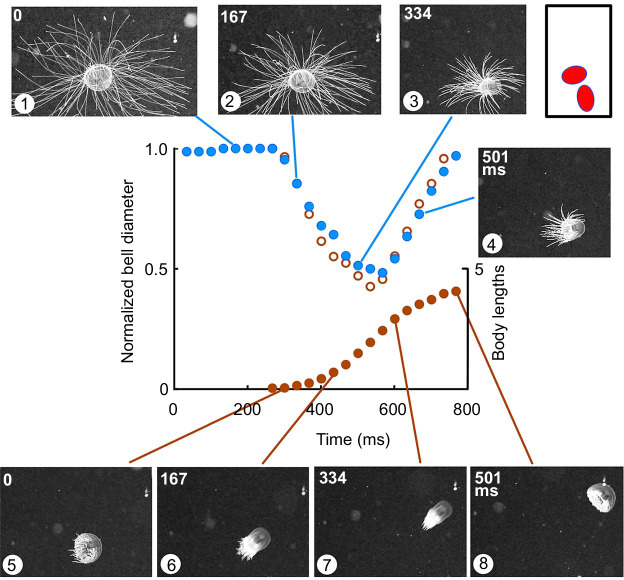
**Swims performed by *Crossota millsae* filmed in its natural environment.** Frames 1–4: frames selected at 167 ms intervals, showing the progression of tentacle retraction during an initial swim. Bell diameter measured at the base of the bell (filled blue circles; left axis), normalized to the resting value. Frames 5–8: frames selected at 167 ms intervals, showing a second swim. Normalized bell diameter (open brown circles; left axis) and forward movement (filled brown circles; right axis) plotted on the same time axis for comparison. Inset (top right): silhouette of bell margin from frames 1 and 5, showing little change in position during the first swim.

Fig. 6 shows one such species, *C.* *millsae*, filmed in its natural environment (Monterey Bay; depth, 1800–3000 m). In frame 1, the animal is seen resting with its tentacles spread ready to trap prey. Tentacle retraction starts as soon the reduction in bell diameter becomes measurable so that by frame 2 the average tentacle length is reduced by 25% (*N*=5). Tentacle withdrawal continues throughout the bell contraction and into the refilling phase. Note, however, that bell contraction brings relatively little forward movement, as is revealed by comparing the silhouettes of the bell margin taken from frames 1 and 5 (Fig. 6, inset, top right). Frame 5 marks the end of the first swim and the start of a second (167 ms after frame 4). The second swim is as powerful as the first, as judged by the change in bell diameter (Fig. 6; open brown circles) but on this occasion the bell travels forward by almost five body lengths (Fig. 6; filled brown circles).

**Fig. 7.**
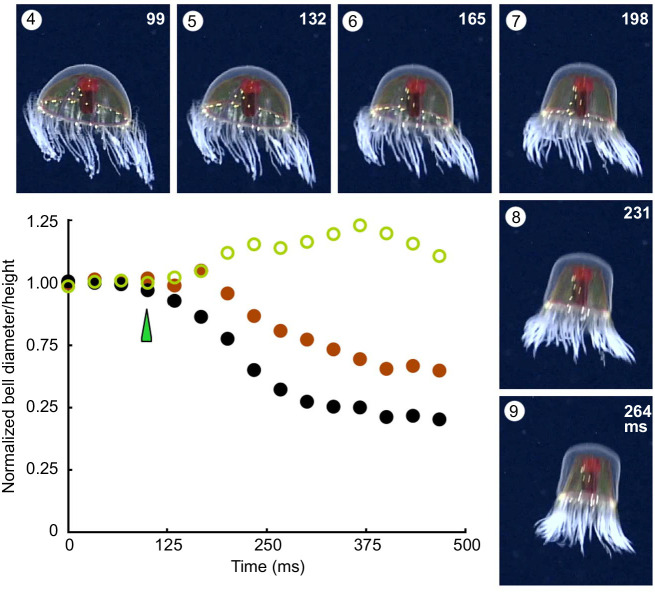
**Change in *C. millsae* bell dimensions during fast swim.** A specimen filmed in its natural environment (depth, 1800–3000 m). Bell diameter (base: filled black circles; mid-bell: filled brown circles) and bell height (open green circles) measured frame by frame and normalized to the values at rest. The onset of the swim (frame 4) is indicated by the green arrowhead. Frame images 4–9 are sequential (33 ms interval) and show that the bell becomes tubular during the swim.

In Fig. 6, the bell contraction developed more slowly than in either *A. digitale* or *C. sericeum*. It lasted 267–300 ms and had a similar refilling time. The second swim started as soon as refilling had completed. In another specimen filmed under natural conditions, the average time between swims was 635 ms (*N*=20). In the specimen shown in Fig. 7, the bell contraction was of a similar strength, as judged by the change in bell diameter, but was slightly longer lasting (434 ms). On this occasion, the animal travelled three body lengths. The size of the animal was not recorded but the species has been described as being up to 28 mm wide (Thuesen, 2003). Its orientation was such that changes in bell height and diameter could be measured sufficiently accurately to reveal a significant delay in the onset of contraction in the mid-bell region.

The bell diameter was measured at a position mid-way up the bell, normalized to its resting value and plotted in Fig. 7 (filled brown circles). Also shown (filled black circles) are the normalized values for the base of the bell and for the bell height (open green circles). At the onset of contraction (green arrowhead) there was a reduction in base diameter while the diameter mid-way up the bell was increased. The bell height also increased. There was a 33–66 ms delay between the onset of contraction at the base and the onset at mid-bell. Frames 4–9 show that the bell became more tubular during the course of the swim as if some of the water contents are forced into the top of the bell. It is not until the mid-bell contracts that there is significant forward movement.

**Fig. 8.**
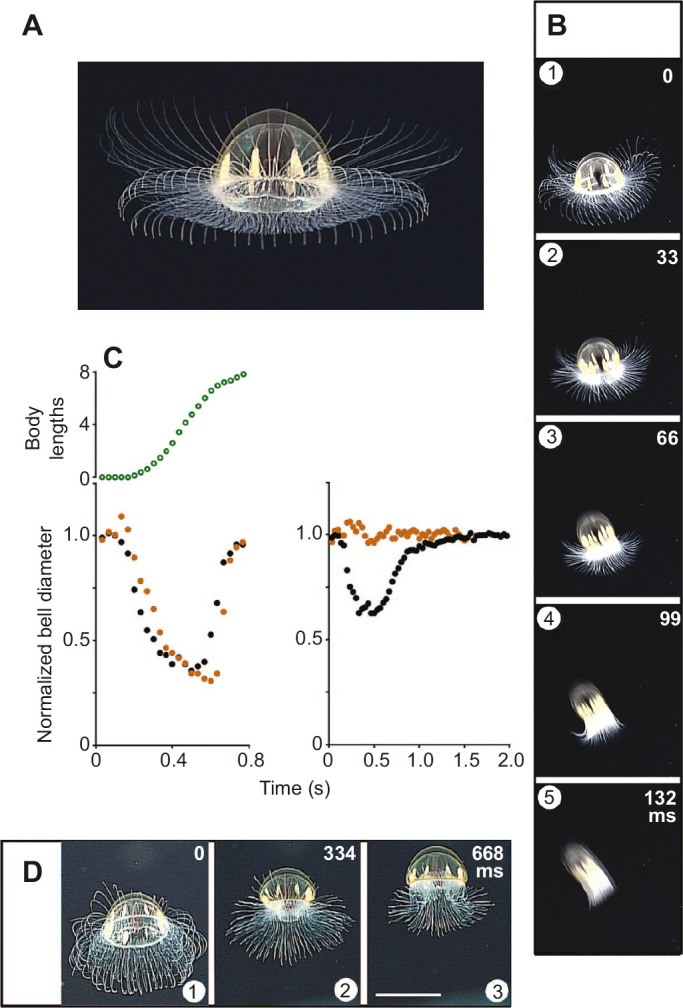
**Fast and slow swimming** ***Benthocodon*** **spp. in their natural environment.** (A) Specimen of *B.* *hyalinus* filmed at ∼3020 m in waters off American Samoa, part of the US Exclusive Economic Zone, at ‘Utu’ Seamount. (B) *B.* *hyalinus* fast swim consecutive frames recorded at 29.97 frames s^−1^. (C) Change in bell diameter during a slow swim (right) and a fast swim (left; mid-bell, filled brown circles; base, filled black circles). Forward movement during a fast swim measured in body lengths (open green circles; animal initially at rest on the sea floor). Data from *B.* *pedunculatus*, at 3500 m (left); *B.* *hyalinus* (right)*.* (D) *Benthocodon hyalinus* slow swim recorded at 29.97 frames s^−1^; non-consecutive frames. A/B and C right/D may be different specimens. Scale bar: 4.25 cm. Video and picture credit: Elliott et al. (2017).

*Crossota millsae*, like other Trachymedusae, is able to perform both fast and slow swims. It resembles *Colobonema* (see Fig. 2E) by using movements of its bell margin for slow swimming. It also regulates these marginal contractions for reorientation as shown in Fig. 3. Other members of the Crossota subclade also exhibit dual swimming. Film records exist for two species of *Benthocodon*, both of which are exceptionally strong swimmers. In *B. hyalinus* (Fig. 8A), tentacle retraction begins at the start of a fast swim but the process is incomplete, tentacle length decreasing by only 30% during the course of the swim (Fig. 8B). Like *B. pedunculatus*, it may be found on the sea floor, apparently foraging for crustaceans and foraminiferans (Matsumoto et al., 1979).

The supplementary video 2 provided by Matsumoto et al. (2020) surveys a group of ten *B. pedunculatus* specimens on or near the ocean floor (depth 3500 m) in Monterey Bay. Towards the end of the sequence, one specimen initiates a slow swim, and this is immediately followed by a fast swim in a close neighbour. The animal travels about seven body lengths with just a single fast swim (Fig. 8C, top left). As with the other members of the Crossota subclade, the contraction develops comparatively slowly, reaching a maximum in ∼400 ms. As with *B. hyalinus*, the contraction spreads all the way up the bell, and in both species the mid-bell contraction appears to lag behind the marginal contraction (Fig. 8C, bottom left; compare with *C.* *millsae*). Relaxation of the margin is faster than the mid-bell and the animal becomes distinctly cone shaped. Fig. 8D shows *B. hyalinus* undergoing a slow swim. As with *Colobonema*, the bell contraction is only half that seen during an escape swim and it is confined to the basal region (see Fig. 8C, right).

## DISCUSSION

The Trachymedusae are a loosely connected group of Hydromedusae (Fig. 1). They share anatomical features, such as eight radial canals and eight gonads, but differences in their nervous systems are immediately apparent. In *A. digitale*, for example, the giant motor axons in the subumbrella musculature are visible under a low-power, dissecting microscope. They run from the bell margin to the bell apex alongside each radial canal and can be as much as 40 µm in diameter. In *C. sericeum*, however, radial canals are present but no giant axons are visible. Electron micrographs of sections of a third member of the Rhopalonematidae, *Pantachogon* sp., also fail to reveal the presence of giant axons (Mills et al., 1985).

Behaviourally, the Rhopalonematidae have a distinctive dual swimming ability. In *A. digitale*, fast swims are clearly specialized for escape whereas slow swims are employed during foraging, the two forms being exhibited at all stages in the life cycle. The material presented here extends our knowledge to other members of the group; but, before discussing the significance of dual swimming, it is necessary to be more precise about its unique nature. We use the term ‘dual swimming’ when fast swimming operates on a distinctly different myoneural basis from regular slow swimming. In *A. digitale*, and we assume other members of the Aglaurinae with giant motor axons, a fast escape swim is a reflex mechanism involving vibration sensors, giant motor axons and contractions of the entire subumbrellar myoepithelium. Slow swims, however, involve pacemaker units and a limited area of myoepithelium on either side of the mid-bell region.

In *C. sericeum* and *P. haeckeli*, fast swims set off by vibrations in the local medium also involve the entire myoepithelium – although not mediated by giant axons – whereas during slow swims, contractions are limited to the region around the margin. The same appears to hold for members of the Crossota clade. Thus, there are distinct differences between dual swimming as uniquely exhibited by the Aglaurinae, and dual swimming as exhibited by the Crossota and the Rhopaloneminae clades. A feature shared by all three clades, and which separates them from other hydromedusae, is the strength of their fast swim. A single swim can transport an individual by five or more body lengths, with a maximum velocity markedly more than values reported by Gladfelter (1973) for other medusae.

The fast swims exhibited by the Crossota clade, although powerful, develop more slowly than those in the Aglaurinae and the Rhopaloneminae. It may be for this reason that the delay between the contraction in the margin and that in the mid-bell region is measurable. It remains to be seen whether there are differences in the way excitation spreads. Meanwhile, the differences in dual swimming between the Aglaurinae and the Rhopaloneminae allow us to pinpoint the appearance of giant-axon-based swimming as an evolutionary event in the lineage leading to the Aglaurinae (blue asterisk in Fig. 1). Other adaptations associated with this transition include the tentacle giant axons, which provide for fast tentacle retraction, and the development of a more streamlined shape (length/diameter ratio, >1).

### Forms of excitability in muscle epithelia

The different forms of dual swimming exhibited by the Rhopalonematidae raise questions about how they are achieved in an electrically coupled myoepithelium. Table 1 is a survey of the physiological basis of swimming reported for a range of different Hydromedusae. Among other things, the table shows that none of the four members of the Halicreatidae, which is a closely related family to the Rhopalonematidae, show two modes of swimming (Mills et al., 1985). In the Ptychogastriidae, another structurally related family, *Tesserogastria musculosa* can swim strongly, but has no motor giant axons and is not able to slow swim (Mills et al., 1985).

**Table 1.**
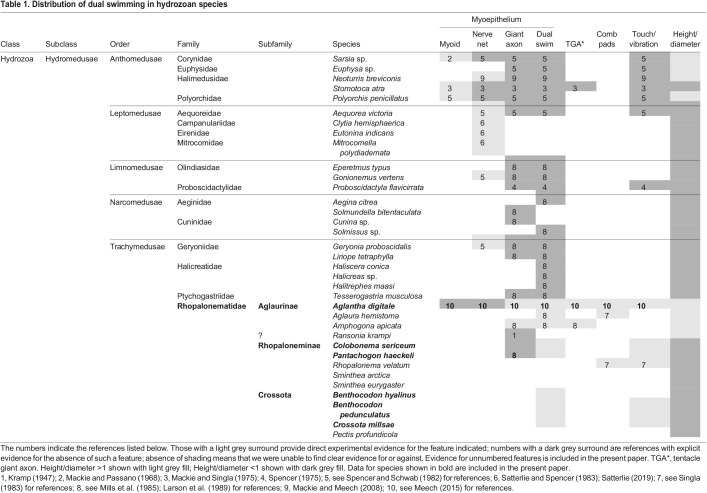
**Distribution of dual swimming in hydrozoan** **species**

Another species worthy of comment is *Ransonia krampi*. Although described as *Aglantha krampi* by Ranson (1932), it was reclassified by Kramp (1947) who called it a “most peculiar species”. Kramp says that it is very like *A. digitale* but the gonads are entirely different in structure and position and “it differs from *Aglantha* in lacking the characteristic bands along the sides of the radial canals”. Having examined a specimen, C.E.M. (unpublished) confirms that *R. krampi* is shaped like *A. digitale* (15 mm high by 10 mm wide) and has a powerful swim. However Kramp's comment regarding the missing “characteristic bands along the radial canals” in *R. krampi* can only be referring to the giant axons that are such an obvious feature of the subumbrella in *Aglantha*. Thus, *Ransonia* has affinities with both *Colobonema* and *Aglantha* in that it is *Aglantha* shaped but may be *Colobonema*-like in neural circuitry. Unfortunately, there are no data for *R. krampi* in GenBank.

Table 1 identifies species in which the spread of excitation within the swimming muscle epithelium depends on motor giant axons, a subumbrella nerve net or myoid conduction. The subumbrella muscle epithelium of *A. digitale* is not directly excitable and does not generate propagating action potentials. In this respect, it is unlike the myoid conduction seen in other hydromedusae, such as *Polyorchis* (Spencer and Satterlie, 1981) or the siphonophores *Agalma* (Chain et al., 1981) and *Chelophyes* (Inoue et al., 2005), in which the myoepithelium is itself electrogenic. In *Neoturris breviconis*, electrical stimulation anywhere on the subumbrellar elicits propagated contractions, but the contractions are accompanied by nerve impulses, and staining with anti-tubulin antibody reveals an embedded nerve plexus (Mackie and Meech, 2008). The subumbrellar myoepithelium in *Aequorea victoria* also has a nerve plexus, and there is evidence for a synaptic input during swimming (Satterlie, 2008). *Clytia hemisphaerica*, *Eutonina indicans* and *Mitrocomella polydiademata* are all reported to have subumbrellar nerve nets (Satterlie, 2019). In *C. hemisphaerica*, much of the nerve net is concerned with feeding behaviour such as mouth pointing and margin folding (Weissbourd et al., 2021). Whether the rest of the nerve net plays a role in swimming in this species is not clear.

Identification of the myoepithelial conduction mechanism in *Colobonema* and *Pantachodon* awaits investigation with intracellular micropipettes; all we know at this stage is that it is distinctly different to the system in *A. digitale*. In *A. digitale*, the myoepithelium is synaptically driven by the giant axons of the motor system. These axons, which are responsible for both fast and slow swimming, have two thresholds and two propagating impulses. The muscle epithelium itself also has two sets of differently voltage-dependent Ca^2+^ channels and hence two thresholds. In the hydrozoa, muscle contraction relies on Ca^2+^ influx and so the high and low threshold Ca^2+^ channels provide a mechanism for producing different strengths of contraction (Mackie and Meech, 1985; Meech, 2015; R.W.M. and G. O. Mackie, unpublished).

### Dual swimming in the Rhopalonematidae

These observations leave us with a number of tantalizing questions. If excitation in the Rhopaloneminae does spread through the subumbrella myoepithelium by myoid conduction, why do slow swims fail to produce more widespread excitation of the bell? Perhaps *Colobonema* muscle resembles *Aglantha* in this respect and Ca^2+^ influx occurs over two separate voltage ranges (R.W.M. and G. O. Mackie, unpublished). This would allow pacemaker-driven slow swims to operate under the threshold for more general excitation. On the other hand, the different strengths of swim may reflect two different nerve inputs, one of them being limited to the marginal area. In this case, the subumbrella nerve plexus concerned may have evolved into the more organized system of giant axons and lateral nerves present in *A. digitale* (Kerfoot et al., 1985). The motor giant axons of *Aglantha* are multinucleate and “not infrequently show bifurcations or give off side branches which flow back into the main axon after wandering separately for some distance” (Mackie, 1990). It is tempting to imagine that they may have evolved by fusion of a more diffuse nerve network.

### Different forms of swimming in Hydrozoa

Table 1 draws attention to the fact that dual swimming is a unique feature of swimming in the Rhopalonematidae. However, we do not wish to imply that the hydromedusae do not exhibit other forms of ‘variable’ swimming. In *Polyorchis penicillatus*, Megill (2002) identifies four ‘gait’ patterns that animals display while fishing or swimming. Mills and Goy (1988) point to the changes in posture exhibited by some Narcomedusae when swimming (see also Raskoff, 2002). In *Aeginura grimaldii*, postural change may hasten escape (Larson et al., 1989), while in some *Solmissus* species, changes in tentacle orientation are associated with differences in swimming speed (Mills and Goy, 1988). In addition, some Narcomedusae alter their speed by changing the strength of their bell contractions (Larson et al., 1989).

The fact is that we do not know the myoneural basis of these gaits, but even if they prove to involve different myoneural pathways, none of them approach the strength of swim exhibited by fast swimming Rhopalonematidae. We therefore distinguish them on this basis. We also exclude mechanisms that simply involve changes in swim frequency if, as seems likely, the underlying myoneural basis remains the same. This includes variations in swim frequency that produce changes in the force of the swim associated with partial filling of the swimming bell, or escape behaviour based on increased swimming frequency. In the hydrozoan *Polyorchis penicillatus*, reductions in light intensity produce an increase in swimming frequency, which may be a means of avoiding predation (Anderson and Mackie, 1977), and there is a similar reaction in response to light in dark-adapted *Sarsia tubulosa* (Passano, 1973). According to Passano (1973), local tentacle contractions may also provide an indirect input to the swim pacemaker system, although whether this is part of an escape reflex is unclear.

Some hydromedusae change the nature of their swims during the course of development. For example, in the leptomedusae *Aequorea victoria* and *Eutonina indicans*, development involves a change from jet swimming, prolate juveniles to larger, more oblate adults that use rowing as a means of propulsion (Weston et al., 2009). These developmental changes are in marked contrast to the different swimming patterns we describe here that are observable in individuals within a timescale of minutes or less.

Finally, we should note the remarkable swimming flexibility exhibited by members of the subclass Siphonophorae, which are not included in Table 1. In *Nanomia bijuga*, for example, the ability to adjust the timing of the contractions of its multiple swimming bells gives it great flexibility in forward movement (Mackie, 1964; Costello et al., 2015). In addition, it is able to redirect its jets and escape swim backwards (Mackie, 1964; Norekian and Meech, 2020). Forward and backward swimming utilize two different motor pathways – dual swimming par excellence but not considered here.

### Functions of dual swimming

All of the Rhopalonematidae described are capable of both fast and slow swimming, and in some cases it is clear that the fast swims serve for evasion. In *A. digitale*, fast swims are rare and always evoked by touch or vibration, but in other species, the situation is less clear cut. For example, *Colobonema*, when swimming in the wild, performs a combination of fast and slow swims (see Fig. 2). It is possible that an initial fast swim is necessary to overcome the drag imposed by the animal's extended tentacles and that the momentum generated is maintained by the slow swims. However, the sensitivity of *Colobonema* to vibration – in the laboratory a relatively slight disturbance in the water surface is enough to evoke a powerful swim – suggests that fast swims may have an additional escape or avoidance function. *Pantachogon* also gives a powerful swim in response to a light touch as, apparently, does *Benthocodon* (see the supplementary material of Matsumoto et al., 2020). In *Aglantha*, the sense organs responsible appear to be ‘hair cell’ structures at the base of the bell (Arkett et al., 1988). We do not yet know whether *Pantachogon*, *Colobonema* or *Benthocodon* possess similar hair cell structures.

The dual swimming in *Colobonema* is the first instance of its being recorded in more oblate jellyfish. When *Colobonema* performs a fast swim, excitation spreads throughout the bell, but during a slow swim, the contraction is restricted to the bell margin. It is possible that slow swimming is a more efficient means of propulsion than fast swimming, because the slower, weaker contractions mean that the water is likely to leave the bell more slowly (see Vogel, 2003).

Another characteristic of slow swimming in *Colobonema* is its high level of maneuverability. Preliminary observations suggest that the marginal contractions are influenced by the animal's orientation; when the animal is pointing upward the contractions are symmetrical, whereas when it is pointing horizontally or downward they are markedly asymmetric. During the course of the swim, as the animal turns, the contraction loses its asymmetry. This suggests that an input from gravity receptors can inhibit contractions in the marginal myoepithelium. Further, it appears that the pacemakers responsible for slow swimming continue to provide an excitatory input for the duration of the long contractile phase of the swim (∼250 ms). This would explain why the non-contracting half of the bell can start to contract so long after the start of the swim, once the inhibition has declined. In some other hydromedusae, such as *Polyorchis*, turning stems largely from an asymmetrical contraction of the velum. According to Gladfelter (1972, 1973), this causes water to leave the bell at an oblique angle, and the animal turns toward that same side. In *Colobonema*, asymmetrical contractions of the velum also contribute to turning. Gladfelter (1973), reports that the combined turning effect of asymmetries in the velum and bell margin, as found in *Gonionemus*, ‘are considerably more effective than the method relying strictly on velar action, which occurs in *Polyorchis*, for the turning radius …. is much smaller’.

In *A. digitale*, the contraction phase of a slow swim lasts for ∼150 ms, each swim arising from a single pacemaker synaptic potential lasting no longer than 60 ms (Meech and Mackie, 1995). In *Colobonema*, the contraction builds over a course of ∼250 ms and may be the result of a series of summated events as it is in *Aequoria victoria* (Satterlie, 1985; 2008). Slow swimming in *Colobonema* and *Aglantha* are similar in that only a limited region of the bell contracts; but, in *Aglantha*, it is a region mid-way up the bell (Meech, 2015), whereas in *Colobonema*, the contraction is restricted to the margin. The myoneural basis of slow swimming in *Aglantha* involves a low-threshold propagating Ca^2+^ spike, but the spike changes in shape as it travels towards the bell apex and provokes stronger contractions in the myoepithelium as it does so (Meech and Mackie, 1995). The details of this mechanism are the subject of a later paper (Meech and Mackie, in preparation).

## Supplementary Material

Supplementary information
