# Supplementary material for: Two swimming modes in Trachymedusae; bell kinematics and the role of giant axons
Source: J Exp Biol. 2021 May 25;224(10):jeb239830. doi: 10.1242/jeb.239830 (PMC8180259; doi:10.1242/jeb.239830)
Supplement: Supplementary information [file jexbio-224-239830-s1.pdf]

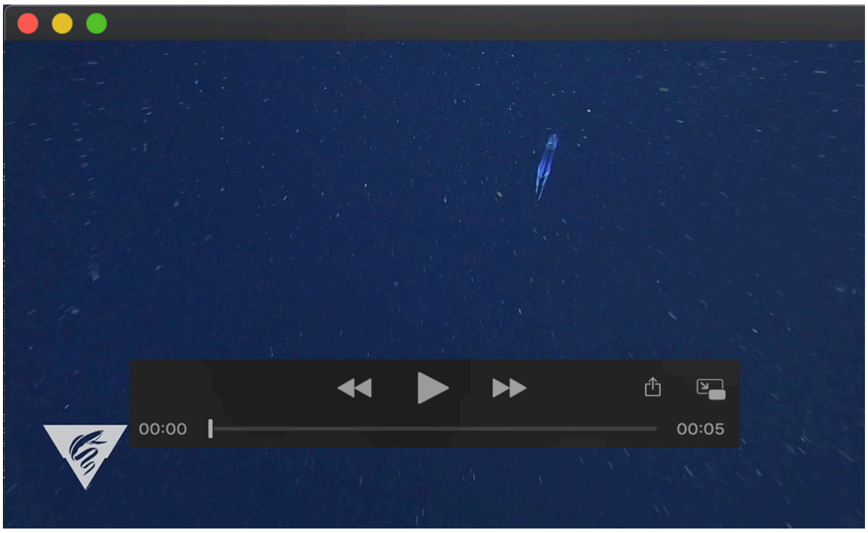

Movie 1. Studied in its natural environment using the ROV *Ventana, Colobonema sericeum* exhibits bursts of powerful swimming as if evading capture. However it is unclear whether the swims are elicited in response to water disturbance created by the ROV or because of the bright light used for filming. As noted by previous authors the animal was likely to lose some or many of its tentacles during high frequency fast swims (see video), which suggests that it is normally a relatively rare occurrence. Although we have not found the released tentacles to be luminescent, they may nonetheless function to distract and discourage potential pursuers (Wrobel and Mills, 1998).
